# Systematic review evaluating randomized controlled trials of smoking and alcohol cessation interventions in people with head and neck cancer and oral dysplasia

**DOI:** 10.1002/hed.25138

**Published:** 2018-03-30

**Authors:** Ellie Shingler, Luke A. Robles, Rachel Perry, Chris Penfold, Andy R. Ness, Steve Thomas, J. Athene Lane, Richard M. Martin

**Affiliations:** ^1^ National Institute for Health Research (NIHR), Bristol Biomedical Research Centre (BRC) Nutrition Theme University of Bristol Bristol United Kingdom; ^2^ Bristol Medical School: Population Health Sciences University of Bristol Bristol United Kingdom; ^3^ Department of Maxillofacial Surgery University of Bristol Bristol United Kingdom; ^4^ Department of Randomised Trials Collaboration University of Bristol, School of Social and Community Medicine Bristol United Kingdom

**Keywords:** alcohol, head and neck cancer, oral dysplasia, systematic review, tobacco cessation

## Abstract

**Background:**

Smoking and alcohol increase the risk of head and neck cancer and affect treatment outcomes. Interventions modifying these behaviors may improve posttreatment outcomes and survival. We systematically reviewed evidence of the effectiveness of smoking/alcohol interventions in head and neck cancer and oral dysplasia.

**Methods:**

The AMED, CINAHL, Embase, MEDLINE, and Web of Science databases were searched for randomized controlled trials (RCTs) of smoking/alcohol interventions in people with head and neck cancer. A qualitative synthesis of the studies was conducted.

**Results:**

Three RCTs were identified: 2 smoking interventions and 1 smoking and alcohol intervention. One intervention, which was comprised of a smoking intervention based on Cognitive Behavioral Therapy and pharmacologic management compared to usual care, reduced smoking prevalence.

**Conclusion:**

Further research is required into the underlying mechanisms that lead to cessation and interventions that include both pharmacological and behavioral therapy. Future RCTs should include suitable control conditions and sufficient power to assess clinical outcomes.

## 1 INTRODUCTION

Head and neck cancers include cancers of the mouth, sinus, larynx, nasopharynx, and oropharynx. It is estimated that 10 000 new cases are diagnosed in the United Kingdom each year^1^ with >550 000 cases per annum diagnosed worldwide.^2^ Survival rates vary according to cancer site. Between 1990 and 2006 the 5‐year survival rate for laryngeal cancer remained the same at 65%. Other forms of head and neck cancer, such as nasopharyngeal and oropharyngeal cancers, have seen an increase in 5‐year survival rates of 10% and 13%‐14%, respectively, due in part to improved diagnosis and treatment.^3^, ^4^ Oral epithelial dysplasia (OED) is an oral lesion preceding the formation of cancerous tissue in 5%‐50% of patients, depending on the type of dysplasia present.^5^, ^6^

It is estimated that 75% of all head and neck cancers are caused by tobacco smoking and alcohol consumption.^7^ One pooled analysis of 17 case‐control studies found the population attributable risk for tobacco and alcohol to be 64% for oral cavity cancer, 72% for pharyngeal cancer, and 89% for laryngeal cancer.^8^ The risk factors for OED are not well understood, although there is evidence that tobacco use and alcohol consumption relate to increased risk of this premalignant condition.^9^, ^10^

As well as increasing the risk of disease, evidence also suggests that smoking and alcohol use can adversely affect cancer treatment outcomes.^11^, ^12^ This may be because people are less likely to respond to treatment if they smoke, resulting in a lower rate of survival^13^ as well as increasing the risk of treatment side effects.^14^ Although the mechanisms behind this are unclear, risk of recurrence and secondary primary tumors has also been reported to be higher in people who continue to consume alcohol after their diagnosis.^15^, ^16^

Evidence of the beneficial effects associated with reduced tobacco and alcohol use has resulted in a growing interest in interventions that target these behaviors and in understanding how these factors can contribute to survival and posttreatment outcomes.^16^

One previous systematic review on smoking interventions in head and neck cancer was identified through an initial scope of the literature.^17^ However, this review included quasi‐experimental designs and people who had recently quit smoking within their search strategy and excluded people with premalignant conditions. No reviews on alcohol interventions in people with head and neck cancer or OED were identified. The purpose of this review was to examine the effectiveness of smoking and alcohol cessation interventions on disease‐related outcomes, quality of life (QOL), and smoking and alcohol cessation in adults with either head and neck cancer or oral epithelial dysplasia. The review has been registered on the Prospective Register of Systematic Reviews (PROSPERO) database of systematic reviews, registration number: CRD42016038237.

## 2 MATERIALS AND METHODS

The full review protocol has been described in detail previously.^18^

### 2.1 Eligibility criteria

Eligibility was defined in terms of population, intervention, control, outcome, and study design. Population: The population included adults who have been diagnosed with either oral epithelial dysplasia or head and neck cancer defined as cancers of the oral cavity, pharynx, larynx, paranasal sinuses, nasal cavity, and salivary glands. Intervention: Studies were included that assessed tobacco or alcohol use reduction or cessation interventions. Comparison: Intervention comparison could include placebo for pharmacological interventions or standard care for behavioral interventions. Studies that compared multiple active intervention arms but did not include a control arm were also included. Outcomes: Studies were included that reported on any of the primary or secondary outcomes of interest. The primary outcomes were disease‐free survival or, for people with oral dysplasia, disease progression to head and neck cancer. Secondary outcomes were disease recurrence, disease progression (in head and neck cancer), QOL, behavioral change, cancer‐specific mortality, second primary cancers (defined as those that originated at least 3‐cm away from the primary site and occurred at least 3 years after the last known recurrence), and all‐cause mortality. Study design: Only randomized controlled trials (RCTs) were included in this review.

### 2.2 Search strategy

The Cochrane Library, AMED, CINAHL, Embase, MEDLINE, and Web of Science databases were searched from their inception to May 23, 2017. The search strategy used in MEDLINE can be seen in Supporting Information Appendix S1. The searches were not limited by earliest date or language of publication. Systematic reviews that were identified through the search were cross‐checked for any studies not found in the original search and the reference lists of all included articles were hand searched for additional studies. The National Institute for Health Research (NIHR) Clinical Trials gateway was searched to identify any trials that had not reached publication stage^19^ and the first 20 pages of Google Scholar were hand searched for any additional articles.

### 2.3 Selection of studies and data extraction

**Figure 1:**
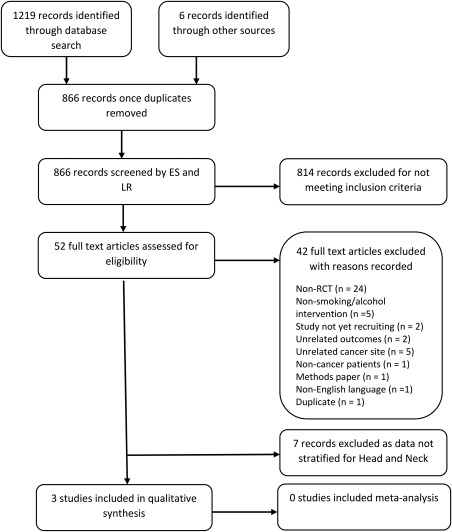
Inclusion flowchart

All titles and abstracts were screened independently by 2 reviewers (E.S. and L.R.). Abstracts that potentially met the inclusion criteria were retrieved and read in their entirety to assess eligibility. Decisions on inclusions and exclusions were recorded (see Figure 1 for flow diagram). Any disagreements were discussed with a third author (R.M.). Two reviewers extracted the data independently using a standardized, pre‐piloted data extraction form created for this review, then compared results for accuracy. Data were extracted on publication information, sample characteristics, intervention type, and results.

### 2.4 Assessment of risk of bias

Risk of bias was assessed using the Cochrane Collaboration's tool,^20^ which has been updated to reflect the current study's review parameter. This tool enabled bias to be assessed on 6 aspects of trial design and reporting where bias may be introduced; sequence generation, allocation concealment, blinding of research participants, blinding of outcome assessors, completeness of outcome data, and outcome reporting. Studies were classified for each criterion as having a low, high, or unclear risk of bias. Any other potential sources of bias identified by the reviewers were also recorded. A copy of the table of assessment criteria used for this study is in Supporting Information Appendix S2.

### 2.5 Data analysis

Between‐group analyses of the main outcomes are presented and a qualitative synthesis of all studies was conducted. Due to the small number of studies found and heterogeneity of the interventions, a meta‐analysis was not deemed appropriate. The behavioral change techniques used by the trials were coded using the Behavioral Change Technique (BCT) Taxonomy, version 1.^21^ The BCT Taxonomy allows for more standardized coding of the behavioral change techniques used in behavioral change interventions, enabling comparison of techniques used between interventions.

## 3 RESULTS

### 3.1 Search results

Details of the selection process and reasons for exclusions are summarized in the inclusion flowchart (Figure 1). The literature search identified 1089 records. Once duplicates were removed, 815 records were screened and assessed against the eligibility criteria. After exclusion of ineligible studies, 10 studies were included in the review. Of these studies, 7 reported results on multiple cancer sites and did not stratify results by cancer type. After contacting the study authors, these were not included in the qualitative synthesis due to unavailability of the head and neck cancer‐specific data. These studies have been summarized in Supporting Information Appendix S2 along with their risk of bias assessments in Supporting Information Appendix S3. The remaining 3 studies reported results for head and neck cancer and were included in the qualitative synthesis.^11^, ^22^, ^23^ The characteristics of these studies are outlined in Table 1.^11^, ^22^, ^23^ The 3 included articles were published between 1993 and 2016 and originated from the United States.

**Table 1:** Characteristics of included studies

Reference,author, year	Population, no. of participants, age, site/lesion type	Intervention	Outcomes	Lengthof follow‐up	Result	Effect estimate
Duffy et al^11^ 2006	184 patients with head and neck cancer, mean age 57 y, 84% men, 90% white	Tailored smoking, alcohol, and depression intervention comprising of CBT and pharmacologic management.	Self‐reported smoking cessation rates. Self‐reported problem drinking rates.	6 mo	Smoking: 47% cessation in intervention compared to 31% cessation in control. Alcohol: 32% improved problem drinking in intervention compared to 30% in control	Smoking: *P* = .048 Alcohol: *P* = .853
Ghosh et al^22^ 2016	14 participants either undergoing treatment for or observation of premalignant lesions or who had received treatment for head and neck cancer >5 y previously. Mean age 60 y.	Financial incentive for smoking cessation	Self‐reported cessation confirmed by exhaled carbon monoxide at 30 d and self‐reported cessation confirmed by a negative urine cotinine assay at 3 mo and 6 mo	6 mo	33.3% cessation in intervention group compared to 0% in control group	N/A due to small sample size
Gritz et al^23^ 1993	186 participants undergoing treatment for cancers of the oral cavity (54.9%), pharynx (6%), and larynx (39.1%). Mean age 58.5 y, 73.7% men, 72.6% white	A physician and dentist‐delivered smoking cessation intervention involving an initial advice session followed by 6 “booster sessions”	Self‐reported cessation confirmed by urine cotinine validation	12 mo	At 6 mo: 64.3% cessation rate in intervention compared to 71% in control. At 12 mo: 63.8% cessation rate in intervention compared to 76.8% in control.	*P* values not reported but the authors state no significant difference between the groups

Abbreviations: CBT, cognitive behavioral therapy; N/A, not available.

### 3.2 Summary of findings

#### 3.2.1 Participants

Altogether, the 3 trials randomized 384 participants with a mean age of 58.5 years, 86% of which were men. One trial included people with either premalignant conditions or head and neck cancer^22^ and 2 included people with head and neck cancer only.^11^, ^23^ One study recruited participants at any point after diagnosis. The mean time since diagnosis was 24 months and ranged from 0 to 282 months.^11^ A second study did not limit the sample based on time because treatment or diagnosis; however, all participants who enrolled were either being treated with ablation and observation of premalignant lesions or had undergone treatment for head and neck cancer more than 5 years previously.^22^ The third study recruited people who had been newly diagnosed with head and neck cancer.^23^

#### 3.2.2 Interventions

All 3 studies assessed a different form of smoking intervention; 1 study using cognitive behavioral therapy (CBT) combined with pharmacotherapy, 1 study was a clinician‐led intervention, and 1 study using financial incentives. One of these also included a CBT and pharmacotherapy‐based alcohol intervention.^11^ The BCTs that were incorporated into each intervention are summarized in Table 2.^11^, ^22^, ^23^ The BCT Taxonomy coding highlights that different techniques were used for each intervention with the exception of “coping skills,” which were used in both the CBT and financial incentive interventions.^11^, ^23^ Participants were followed up for 6 months in 2 studies^11^, ^22^ and 12 months in the third study.^23^

**Table 2:** Behavioral Change Technique Taxonomy codes

Reference, author, year	Taxonomy codes
Duffy et al^11^ 2006	1.1 (goal setting); 1.2 (coping skills); 2.3 (self‐monitoring); 4.1 (social skills training); 4.2 (analyzing antecedents); 11.1 (pharmacological support)
Ghosh et al^22^ 2016	10.2 (material reward); 10.8 (outcome incentive)
Gritz et al^23^ 1993	1.2 (tobacco withdrawal); 1.3 (target quit date); 1.5 (booster session review); 1.8 (contract); 3.1 (staff support); 4.1 (tips on how to quit); 5.1 (risks and benefits); 15.1 (statement of confidence)

#### 3.2.3 Comparison

Two studies used some form of usual care as the comparison group. This was described as “enhanced” usual care^11^ or “standardized” usual care.^23^ “Enhanced usual care” within the smoking and alcohol trial included brief counseling for smoking and/or alcohol problems as required along with a handout for local/state resources for smoking and alcohol cessation^11^ Standardized usual care was comprised of provision of information on the risk of smoking and the benefits of cessation along with advice to stop smoking.^23^ The third study used an “information only” group as the control and both the intervention and control groups were entitled to free enrollment in smoking cessation classes.^22^

#### 3.2.4 Outcomes

None of the included studies reported on the clinical primary outcomes of interest (ie, disease‐free survival in head and neck cancer or disease progression in oral epithelial dysplasia). The main outcome variable in all 3 studies was behavioral change measured by self‐reported smoking status^11^, ^22^, ^23^ or alcohol consumption rate.^11^ Smoking status was confirmed by urine cotinine assay in 1 trial^23^ and either urine cotinine or exhaled carbon monoxide validation measures in another.^22^ One study also reported on QOL as measured by the 12‐item Short Form Health Survey, although no difference was observed either between groups or between baseline and 6 month follow‐up.^22^

#### 3.2.5 Effectiveness of smoking interventions

Only 1 intervention (n = 184) was found to have an effect on smoking status. This intervention comprised of a tailored smoking, alcohol, and depression intervention that used both nurse‐delivered CBT and pharmacologic management in the form of nicotine replacement therapy and/or bupropion for smokers, including the patch (n = 20), gum (n = 4), inhaler (n = 5), bupropion (n = 6), paroxetine (n = 6), fluoxetine (n = 1), and sertraline (n = 1).^11^ Authors found a difference in smoking cessation rates in the intervention group (47%) compared with the control group (31%; *P* < .05). Although not powered for subgroup analysis, their results showed that participants treated for comorbid smoking and depression (n = 64) had higher smoking cessation rates than those in usual care (51% compared to 17%, respectively).

A second study (n = 14) used financial incentives for smoking cessation in which participants received $150 if smoking cessation was confirmed at each follow‐up time point (30 days, 3 months, and 6 months). However, low enrollment rates (24 of 114) and a high attrition rate (71%) made it inappropriate for authors to conduct a statistical analysis of the results. This led authors to conclude that financial incentives were ineffective as a smoking intervention in people with head and neck cancer.^22^

The third study (n = 186) used a physician‐delivered and dentist‐delivered smoking cessation intervention, which comprised of an initial advice session and 6 booster sessions. The initial advice session included information on the risks of continued smoking and the benefits of cessation, a discussion of the subject's receptivity to quitting, a statement of confidence in the subject's ability to stop, 3 self‐help booklets, a discussion of tobacco withdrawal, a discussion to determine a target quit date, and an affirmation of continuing provider support during the follow‐up period. The booster sessions consisted of advice tailored to the subject's current smoking status. At the 12‐month follow‐up, continuous abstinence rates were higher in the control group (76.8%) than in the intervention group (63.8%). Authors do not report on the *P* values for these results but state that no difference was found between smoking cessation rates in the intervention and control groups.^23^

#### 3.2.6 Effectiveness of alcohol interventions

No reduction in problem drinking, as defined by the Alcohol Use Disorder Identification Test,^24^ was found in the tailored smoking, alcohol, and depression intervention. In the intervention group, 32% improved problem drinking compared to 30% in the control group.^11^

#### 3.2.7 Risk of bias

The risk of bias assessments for each study are summarized in Table 3.^11^, ^22^, ^23^ The 2 studies that did not report differences between intervention and control groups were deemed to have a high risk of bias on one or more criteria.

**Table 3:** Assessment of risk of bias of included studies

Reference, author,year	Sequencegeneration	Allocationconcealment	Blinding ofparticipants andpersonnel	Blinding ofoutcomeassessors	Outcomedatacompleteness	Outcomereporting	Othersources ofbias
Duffy et al^11^ 2006	Unclear	Unclear	Low	Unclear	Low	Low	Unclear
Ghosh et al^22^ 2016	Low	Unclear	Low	Unclear	High	Low	High
Gritz et al^23^ 1993	Unclear	Unclear	Low	Unclear	Low	Low	High

Within the financial incentive intervention,^22^ the risk of attrition bias was high, the imbalance in the number of dropouts between groups was found to be >10%. It also included a small sample size, which may not have been representative of the sample population as only those who were being treated for premalignant conditions or had finished their treatment over 5 years previously enrolled in the trial.^22^

In the physician‐delivered and dentist‐delivered interventions, the design used a standardized version of usual care for the control, as they found “advice‐giving practices varied widely among providers from none at all to inquiries and warnings about smoking behavior at every visit.”^23^ There is potential for contamination of the control group as it was delivered by the same physicians as the intervention. This might be expected to reduce the difference between the randomized groups.

Although the combined CBT and pharmacological intervention trial reported a reduction in smoking status, this study was deemed to have an unclear risk of bias on the following criteria: random sequence generation, blinding, and allocation concealment. In addition, a different measure of smoking status was used at baseline and follow‐up (smoking status in the last 6 months at baseline compared to current smoking status at follow‐up). Unlike the 2 other studies included in the synthesis, biochemical verification of smoking status was not used, so the results rely on self‐reported data, which is at a higher risk of bias.

## 4 DISCUSSION

This review synthesizes the results of smoking and alcohol interventions in trials with people with head and neck cancer and premalignant dysplasia in order to examine the effectiveness of these interventions on behavioral change, QOL, and clinical outcomes. These results show that few trials have been conducted into smoking and alcohol interventions for people with head and neck cancer and oral dysplasia.

Of the 3 studies included in the review, only 1 study, using nurse‐led CBT alongside pharmacologic management, was found to have an effect on smoking status. This study relied on self‐report measures and was deemed to have an unclear risk of bias. These results are consistent with previous findings into smoking cessation interventions in head and neck cancer.^17^ The review on the effectiveness of smoking cessation interventions in cessation rates in patients with head and neck cancer included 2 of the 3 interventions we report on^11^, ^23^ as well as 1 study with a quasi‐experimental design, which tested a brief intervention with pharmacologic management.^25^ As with our review, the only intervention the reviewers identified that resulted in a difference between groups was the CBT and pharmacologic intervention study.^11^ Both systematic review results are also consistent with the findings of smoking intervention studies conducted within the general population. One Cochrane review showed that combining pharmacotherapy and behavioral therapy increased cessation compared to usual care.^26^

Although the use of financial incentives has been found to boost cessation rates in some populations,^27^ the low recruitment and high attrition rates in the financial incentive intervention^22^ may indicate that financial reward is not a strong incentive in people with premalignant conditions or those who have completed treatment for head and neck cancer. Further research could help to identify what incentives, financial or otherwise, incite behavioral change in this population group. Using this kind of formative research to increase our understanding of the underlying mechanisms that result in change in smoking status for people with head and neck cancer and oral dysplasia, may result in the design of more successful interventions.^28^ The fact that this trial only recruited people who were being treated for premalignancy or had completed treatment >5 years previously is also of interest. The diagnosis of cancer is a health event that could be used as a “teachable moment” to prompt or facilitate desired behavioral change.^29^ Exploring the implementation of interventions at alternative time points on the head and neck cancer treatment pathway, such as diagnosis or commencement of treatment, could help identify the most effective teachable moments.

This review highlights the potential for contamination of the control group when standardizing “usual care.” Contamination can occur if healthcare providers who routinely provide support over and above that of the trial's standard care protocol continue to do so during the trial. Conversely, standardization may actually increase the level of support provided by other healthcare providers. Both of these scenarios can dilute the perceived effectiveness of interventions. This issue can be further compounded if the same healthcare professionals are delivering both the control and intervention conditions. Techniques for minimizing this risk of bias, such as the use of cluster randomization,^30^ could be considered for future trial design, although these may increase other risks of bias, for example, selection bias.^31^

Six further smoking cessation interventions, reported in 7 papers, were identified through the literature search. However, these were not included in the qualitative narrative, as the results were not stratified by cancer site. Similar to the studies included in the review, no significant effects were reported on abstinence rates and risk of bias was deemed as unclear or high (see Supporting Information Appendices S2 and S3). Although the benefits of smoking cessation and reducing alcohol intake for cancer survivors are well documented in observational research, few trials have been conducted in patients with cancer^32^ and it is unclear how cessation interventions vary depending on cancer type.^33^ It would, therefore, be of benefit for future studies that recruit participants with differing cancer types to report the differences in cancer‐site‐specific cessation rates.

The main strengths of our review are the systematic and detailed search strategy used to find eligible studies and the use of 2 reviewers to screen and extract the data. Including people with head and neck cancer and premalignant conditions in our search criteria allowed us to include interventions that recruited both participant groups. As discussed previously, our narrative and conclusions are limited by the exclusion of a number of interventions based on the inability to extract head and neck specific data.

In summary, the paucity of data along with the unclear reporting and risk of bias in trials conducted to date means we are unable to draw any conclusions on the effectiveness of smoking and alcohol interventions in people with head and neck cancer. As there are no data from RCTs on the effects of smoking and alcohol interventions on clinical outcomes, such as progression‐free or disease‐free survival, long‐term follow‐up to explore the impact of interventions on these outcomes is required. Our review highlights that further research is required into smoking and alcohol interventions in patients with head and neck cancer and the effects these interventions have on disease‐free survival and disease progression. Further studies could focus on formative research into the underlying mechanisms that lead to successful cessation in this population group, suitable control conditions/trial design, and interventions that include both pharmacological and behavioral therapy.

## ACKNOWLEDGMENTS

The authors thank Cath Borwick, Information Specialist at the University of Bristol, for her contribution to the search strategy.

## FUNDING INFORMATION

This systematic review is funded by the National Institute for Health Research (NIHR) Bristol Nutritional Biomedical Research Unit based at University Hospitals Bristol NHS Foundation Trust and the University of Bristol. The views expressed in this publication are those of the authors and not necessarily those of the NHS, the National Institute for Health Research or the Department of Health. R.M.M. is recipient of a Cancer Research UK Programme Grant – the Integrative Cancer Epidemiology Programme (C18281/A19169).

## DISCLOSURE STATEMENT

The authors declare that they have no conflict of interest.

## Supporting information

Additional Supporting Information may be found online in the supporting information tab for this article.

Supporting Information Appendix S1

Supporting Information Appendix S2

Supporting Information Appendix S3
