# Supplementary material for: Systematic review evaluating randomized controlled trials of smoking and alcohol cessation interventions in people with head and neck cancer and oral dysplasia
Source: Head Neck. 2018 Mar 30;40(8):1845–53. doi: 10.1002/hed.25138 (PMC6120449; doi:10.1002/hed.25138)
Supplement: Supplementary file 2 — Supporting Information Appendix S2 [file HED-40-1845-s002.docx]

**Appendix 2: Results table for papers not included as they do not report on HNC participants separately**

| **Reference**  (author, year) | **Population**  (n, age, site/lesion type) | **Intervention** | **Outcomes** | **Length of follow-up** | **Result** | **Effect Estimate** |
| --- | --- | --- | --- | --- | --- | --- |
| Ostroff, 2014 | 185 cancer patients, 17 (9%) of which were head and neck. Mean age 55.9. 47% men. 87% white. | A “scheduled reduced smoking” strategy delivered pre-hospitalisation for surgical treatment | 7-day point prevalence abstinence confirmed by salivary cotinine validation. | 6 months | 32% quit rate in both intervention and best practice control groups. | OR =1.03, 95% CI: 0.52 – 2.01 |
|  |  |  |  |  |  |  |
| Schnoll, 2003 | 432 cancer patients, 16 (3.7%) of which were head and neck. 34.3% men. 72.5% white. | A physician-based smoking cessation intervention | Self-reported 7-day point prevalence abstinence | 12 months | 13.3% quit rate in intervention arm compared to 13.6% in usual care | P=0.52 |
|  |  |  |  |  |  |  |
| Schnoll, 2005 | 109 head and neck and lung cancer patients, 32 (29%) of which had head and neck. 54% men. 90% white | CBT based intervention | Self-reported quit rates | 3 months | 43.2% in CBT group compared to 39.2% in general health education control | P= 0.8346 |
| Schnoll, 2010 | 246 cancer patients  Mean age 54.8. Proportion of head and neck not specified | Treatment with bupropion in addition to transdermal nicotine and counselling. | 7-day point-prevalence abstinence  confirmed by breath CO | 6 months | 18.4% abstinence rate in intervention compared to 17.4% in placebo | OR = 1.36, 95% CI: 0.38–4.81 |
| Schnoll, 2011  (reporting same study results as above) |  |  |  |  |  |  |
| Stanislaw, 1994 | 26 hospitalised surgical cancer patients 21 (81%) of which had head and neck. 27% men. 96% white. | Nurse delivered smoking cessation intervention on hospitalised patients | Abstinence confirmed by salivary cotinine validation. | 5 weeks post-discharge | 75% abstinence rate in the intervention compared to 42.9% in usual care. | P < 0.1 |
| Wakefield, 2004 | 137 patients with mixed cancer sites of which 17% had head and neck. Mean age 52. 62% men. | Motivational interviewing intervention | 7 day point prevalence quit rates confirmed by either a urine sample for cotinine  analysis or a breath carbon monoxide | 6 months | 7% abstinence rate in intervention compared to 6% in control | P = 1 |
