# Supplementary material for: Systematic review evaluating randomized controlled trials of smoking and alcohol cessation interventions in people with head and neck cancer and oral dysplasia
Source: Head Neck. 2018 Mar 30;40(8):1845–53. doi: 10.1002/hed.25138 (PMC6120449; doi:10.1002/hed.25138)
Supplement: Supplementary file 3 — Supporting Information Appendix S3 [file HED-40-1845-s003.docx]

**Appendix 3: Assessment of Risk of Bias of studies that did not report separately on head and neck**

| **Reference**  (author, year) | **Sequence Generation** | **Allocation Concealment** | **Blinding of participants and personnel** | **Blinding of outcome assessors** | **Outcome Data Completeness** | **Outcome Reporting** | **Other Sources of Bias** |
| --- | --- | --- | --- | --- | --- | --- | --- |
| Ostroff, 2014 | Low | Low | Low | Unclear | Low | Low | Unclear - ? true control |
|  |  |  |  |  |  |  |  |
| Schnoll, 2003 | Low | Low | Low | Unclear | Low | Low | Unclear - ? true control |
|  |  |  |  |  |  |  |  |
| Schnoll, 2005 | Unclear | Unclear | Low | Unclear | Low | Low | Unclear – used only self-report |
| Schnoll, 2010 | Low | Low | Low | Unclear | Low | Low | Low |
| Schnoll, 2011  (reporting same study results as above) |  |  |  |  |  |  |  |
| Stanislaw, 1994 | Unclear | Unclear | Low | Unclear | Unclear | Low | High – small sample |
| Wakefield, 2004 | Unclear | Unclear | Low | Unclear | High | Low | High – small sample and low recruitment rate |
